# Neighborhood Environments and Cognitive Decline in Middle and Old Age in China: Gender and Age Variations

**DOI:** 10.1093/geroni/igab046.946

**Published:** 2021-12-17

**Authors:** Ye Luo, Xi Pan, Lingling Zhang

**Affiliations:** 1 Clemson University, Central, South Carolina, United States; 2 Texas State University, San Marcos, Texas, United States; 3 University of Massachusetts Boston, Boston, Massachusetts, United States

## Abstract

Older adults are more vulnerable to neighborhood physical and social conditions due to longer exposure, increased vulnerability, changing spatial use, and a greater reliance on access to community sources of integration. Previous research has demonstrated an association between neighborhood environments and cognitive function in older adults. However, most studies were cross-sectional, focused on western countries, and did not examine potential moderating factors. This study examined gender and age variations in the relationship between neighborhood environments and cognitive decline in middle and old age in a developing country that is experiencing rapid population aging and rising prevalence of Alzheimer’s disease and related dementias. Using data from a nationally representative sample of adults aged 45 years and older from the three waves of China Health and Retirement Longitudinal Study (CHARLS 2011-2015), this study estimated multilevel growth curve models for the effects of neighborhood environments on cognitive decline separately for men and women and for those aged 45 to 64 and those aged 65 and above. It showed that the cross-sectional effect of outdoor facility and longitudinal effect of handicapped access were more significant for men, but the cross-sectional effect of community social participation and longitudinal effects of raining days, number of disasters, employment service, and community SES were more significant for women. The cross-sectional effect of infrastructure advantages and longitudinal effects of employment service and old age income support were more significant for adults aged 65 and over. These findings suggest that community-level interventions may be more beneficial for older women.

